# Interpretable machine learning integrating intra-/peritumoral CT radiomics and serum indicators for predicting poorly differentiated esophageal squamous cell carcinoma

**DOI:** 10.3389/fonc.2026.1839893

**Published:** 2026-09-04

**Authors:** Jun Chen, Jiqiang He, Xiaojiao Zhang, Xiaolin Tang, Ming Yang, Fei Wang

**Affiliations:** 1 Department of Radiology, Luzhou People’s Hospital, Luzhou, China; 2 Department of Pathology, Luzhou People’s Hospital, Luzhou, China

**Keywords:** esophageal cancer, machine learning, pathological differentiation, radiomics, SHapley additive exPlanations

## Abstract

**Purpose:**

To develop an interpretable ensemble learning model integrating multiple machine learning algorithms, intratumoral and peritumoral CT radiomics, and serum biomarkers for predicting poorly differentiated esophageal squamous cell carcinoma (ESCC).

**Methods:**

This retrospective study enrolled 261 ESCC patients, who were randomly allocated to training (n=183) and validation (n=78) cohorts. Radiomics features were extracted from intratumor, peritumoral 0.3 cm and intra-peritumoral 0.3 cm areas of enhanced CT arterial phase. Serum neutrophils (NEU) and alkaline phosphatase (ALP) were collected. Following feature dimensionality reduction, ensemble radiomics score (ENs) was calculated for each region. Seven individual machine learning models and an ensemble model (ENML) were subsequently constructed. Model performance was assessed using the area under the curve (AUC), confidence interval (CI) and decision curve analysis (DCA), while interpretability was evaluated via SHAP, correlation, and restricted cubic spline (RCS) analyses.

**Results:**

In the validation cohort, ENML achieved the highest AUC (0.799), F1 score (0.810), recall (0.870), and Brier score (0.166), and demonstrated clinical net benefit across threshold probabilities ranging from 10% to 75%. Bootstrap resampling with 1,000 iterations confirmed its stable performance in the full cohort (AUC: 0.849, 95% CI: 0.790–0.891). Spearman correlation analyses revealed strong positive associations between intratumoral and peritumoral radiomic features (r = 0.54, 0.49, and 0.51, respectively; all P < 0.001). RCS analyses identified significant nonlinear relationships between intratumor, intra-peritumoral 0.3 cm, ENs, and ALP levels and the increased incidence of poorly differentiated ESCC (Overall P < 0.05; Nonlinear P < 0.05). Additionally, both ALP and NEU exhibited significant inflection point effects in predicting poorly differentiated ESCC. SHAP analyses identified the IntraPeri3mm_wavelet.LLL_glszm_SmallAreaLowGrayLevelEmphasis and SVM as the primary contributors to the fusion model.

**Conclusions:**

The interpretable ENML model exhibits favorable discriminative capability for predicting poorly differentiated ESCC; however, further multicenter external validation is warranted to confirm its generalizability.

## 1 Introduction

Esophageal cancer is the seventh most common and sixth leading cause of cancer death globally (1). Esophageal squamous cell carcinoma (ESCC) accounts for 90% of cases, with a rising incidence and a persistently low 5-year survival rate of 10–20% (2, 3). The degree of pathological differentiation of ESCC is a critical biomarker of tumor heterogeneity; well-differentiated ESCC correlates with better prognosis than moderately or poorly differentiated types (4). To be more specific, poor tumor differentiation indicates that the biological characteristics of the tumor are more “aggressive”, including changes in cell structure, high expression of transcription factors, matrix remodeling, vascular heterogeneity, and changes in the inflammatory microenvironment (5, 6). However, in clinical practice, relying solely on endoscopic brushing or small biopsy specimens often leads to underestimation or misdiagnosis of ESCC pathological grading. Additionally, the pathological differentiation degree of ESCC requires comprehensive postoperative evaluation, which delays preoperative treatment decisions (7).

Notably, the contrast-enhanced computed tomography (CT) can provide additional supplementary information regarding tumor vascularity and internal perfusion heterogeneity (8). In recent years, several studies have shown that CT-based radiomics has emerged as a robust method to quantify the tumor microenvironment and intratumoral heterogeneity of ESCC (9–12). For example, Xie C et al. (10) has demonstrated that CT radiomic features derived from different regions can serve as potentially effective biomarkers for predicting the prognosis. Furthermore, Yan T et al. (11) included 205 ESCC to establish a predictor of overall survival in ESCC patients, and the nomogram owned an area under curve (AUC) of 0.837 in the test set. Shi L et al. (12) included 105 ESCC patients to investigate the pathological complete response after neoadjuvant immunotherapy plus chemoradiotherapy for ESCC, and the extreme gradient boosting classifiers (XGBoost) model achieved an AUC of 0.80 in test set. Moreover, Wang Y et al. (4) found that iodine concentration was a biomarker for distinguishing poorly differentiated from non-poorly differentiated ESCC by analyzing spectral CT imaging parameters of 191 ESCC patients, with an AUC of 0.729. Moreover, Zhu M et al. (13) included 200 ESCC patients, aiming to integrate the intratumoral and peritumoral 0.3cm CT radiomics, and to jointly use machine learning to evaluate the overall survival of ESCC patients who received radical radiotherapy and chemotherapy. However, most previous CT radiomics studies have primarily focused on the survival rates of ESCC, with limited research on predicting pathological differentiation in ESCC. Moreover, it remains unclear whether CT radiomics of peritumoral 0.3cm region provides any predictive benefit for preoperative assessment of ESCC pathological differentiation.

Importantly, different machine learning algorithms have different characteristics and applicability. Machine learning models, including random forest (RF), logistic regression (LR), k-nearest neighbor (KNN), XGBoost, support vector machine (SVM), Bayes, neural network (NNet), and others, have been demonstrated potential value in ESCC. However, most of the machine learning models for ESCC are in a “black box” state and lack interpretability analyses.

From a biological perspective, the complementary value between the intratumoral and peritumoral regions can be explained by their distinct pathophysiological significance (13). Radiomic features within the tumor primarily describe the intratumoral heterogeneity of ESCC, such as necrosis, cell density, and vascular irregularity, which are closely associated with tumor pathological grading and aggressiveness (14). In contrast, radiomic features surrounding the tumor capture subtle changes in the adjacent stroma, including inflammatory cell infiltration, fibroblast activation, micrometastasis, and early lymphovascular invasion (14, 15). However, it is unclear whether the combined intratumoral and peritumoral CT radiomics have a beneficial effect for predicting poorly differentiated ESCC, and the potential relationship between intratumoral and peritumoral CT radiomics still needs further investigation.

Hence, we are primarily interested in whether peritumoral 0.3 cm radiomics can add enhanced value to intratumor of ESCC, and to explore the relationship between intratumoral and peritumoral radiomics using SHapley additive exPlanations (SHAP) and correlation analyses. More specifically, this study aims to develop an interpretable ensemble learning model for predicting poorly differentiated ESCC based on different machine learning, intratumoral and peritumoral CT radiomics, and serum indicators.

## 2 Materials and methods

### 2.1 Subjects

This retrospective study was approved by the Ethics Committee of our institution (LKT202405016), and the patients’ informed consent was waived.

Between January 2016 to October 2024, we searched all ESCC patients who received pathological diagnosis in our hospital. Inclusion criteria included: (a) According to the 8th edition of the American Joint Committee on Cancer (AJCC) (16), all patients’ pathological differentiation were categorized as well-, moderately-, and poorly-differentiated ESCC, and the pathological staging of all patients was clear; (b) Preoperative chest contrast-enhanced CT conducted within one month, and complete arterial phase CT images were available. Exclusion criteria included: (a) having undergone preoperative anticancer treatment; (b) poor quality of imaging data; and (c) history of any other concurrent malignancies.

**Figure 1:**
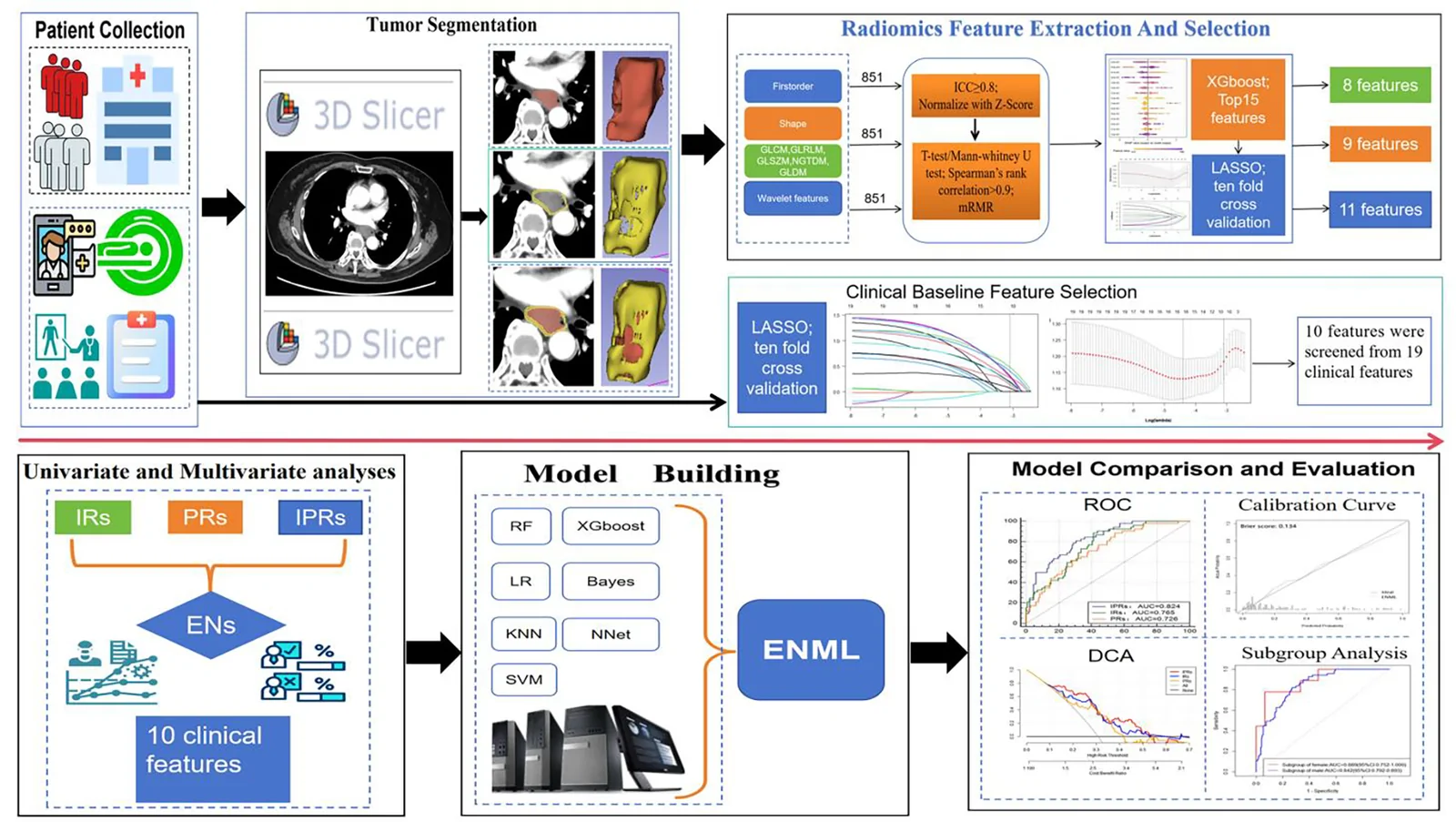
The workflow and design of the study.

Ultimately, 261 ESCC patients including 105 well-, 80 moderately-, and 76 poorly-differentiated ESCC were analyzed in this study, who were randomly divided into a training cohort (n=183) and a validation cohort (n=78) in a ratio of 7:3. **Supplementary Figure 1** shows the flowchart of patient screening. The workflow of the study is summarized in **Figure 1**.

### 2.2 Clinical baseline parameters

We collected basic information for all patients, including gender, and age, along with clinical baseline parameters such as tumor location, length, and maximum diameter of ESCC. Preoperative clinical laboratory indicators comprised white blood cell count, neutrophil (NEU), alanine aminotransferase (ALT), aspartate aminotransferase (AST), alkaline phosphatase (ALP), gamma-glutamyl transferase (GGT), among others. To convert continuous preoperative laboratory data into binary variables, we performed receiver operating characteristic (ROC) curve analysis based on the optimal cut-off value defined by the maximum Youden index, in accordance with previous literature (17). The detailed laboratory parameters and corresponding optimal cut-off values are presented in **Supplementary Table 1**.

### 2.3 CT scanning techniques

Before undergoing CT scanning, all subjects were required to fast and avoid water for six hours. All subjects underwent chest enhanced CT scans on GE Revolution 256 CT (General GE, USA) and GE Lightspeed 16 CT (General GE, USA). Enhanced CT scanning was performed after administering a standard dose (1.5ml/kg) of iohexol injection by using a high-pressure injector, and the concentration was 300mg/ml, injection flow rate was 3-4ml/s. CT arterial phase scan was performed after 18-30s, and then inject 20ml of physiological saline at the same flow rate. The detailed scanning parameters are listed in **Supplementary Table 2**.

### 2.4 Image preprocessing and tumor segmentation

Before exporting the CT images from the PACS system, we masked all the personal information of the ESCC patients (name, phone number and address), and re-named each patient using a numerical format (1, 2, 3,…). Image preprocessing was necessary to standardize radiomics feature extraction. Before the segmentation, in order to eliminate the influence of different devices and scanning parameters on the image, all CT arterial phase image was resampled to a uniform voxel size of 1mm × 1mm × 1mm. Furthermore, normalization and gray-level discretization (bin width =25) were used to standardize the CT image (18).

The segmentation of ESCC was conducted using 3D Slicer software (version 5.0.2, https://www.slicer.org/) on CT arterial phase images to create tumor masks. Tumor segmentation was manually achieved by two radiologists (F.W. and J.C., with 10 and 13 years of CT experience, respectively) on the preprocessed CT images, resulting in a 3D volume of interest (VOI). Subsequently, the peritumoral 0.3 cm region was automatically expanded by using the “Margin” module of 3D Slicer, and surrounding blood vessels, bones, and lung tissue areas were removed.

Additionally, to assess the consistency of the features, 30 patients were randomly chosen and their segmentation was independently redone by Reader 1 (J.C.) and Reader 2 (M.Y. with 25 years of CT experience) after a month. The intraclass correlation coefficient (ICC) was calculated to assess the repeatability and stability of the radiomics parameters.

### 2.5 Radiomics feature extraction and selection

Radiomics features were extracted using PyRadiomics, an open-source Python package (https://pyradiomics.readthedocs.io/en/latest/). A total of 2553 radiomics features were obtained from three VOIs on the arterial phase: intratumoral, peritumoral 0.3 cm, and intra-peritumoral 0.3 cm. In order to avoid data leakage, feature filtering and model construction are only performed on the training cohort, and the validation cohort data remains “invisible” throughout the entire model development and feature selection process.

Prior to feature selection, radiomics features were normalized, and only those with an intraclass correlation coefficient (ICC) greater than 0.80 were retained (19). Dimensionality reduction was performed through the following steps: (a) identifying features with significant differences between groups using the t-test for normally distributed features and the Mann–Whitney U test for non-normally distributed features (P ≤ 0.05); (b) identifying highly correlated features using Spearman’s rank correlation coefficient (Spearman’s rank correlation > 0.9); (c) applying the maximum relevance and minimum redundancy (mRMR) method to remove redundant and irrelevant features while maximizing correlation with the classification variable; (d) ranking the remaining features using the XGBoost machine learning algorithm and retaining the top 15 features with the highest contribution based on SHAP values (20); and (e) performing feature selection on the remaining features using the least absolute shrinkage and selection operator (LASSO) with ten-fold cross-validation (21).

Based on the non-zero coefficients and weights of the retained features, radiomics scores were calculated for the intratumoral (IRs), peritumoral (PRs), and intra-peritumoral (IPRs) regions of ESCC on the CT arterial phase. Additionally, ensemble scores (ENs) were constructed by combining IRs, PRs, and IPRs via logistic regression.

### 2.6 Model building and evaluation

In the training cohort, we sequentially applied univariate and multivariate logistic regression to identify independent risk factors based on clinical characteristics and ENs. Independent risk factors with a p-value < 0.05 were then used for subsequent model development. Seven machine learning models—RF, LR, KNN, XGBoost, SVM, Bayes, and NNet—were constructed in the training cohort. Additionally, we built an ensemble model (ENML) by performing simple logistic regression on the predicted probabilities from all machine learning models (22, 23). We conducted a systematic hyperparameter optimization for seven machine learning models, mainly employing the methods of grid search and 10-fold cross-validation. The detailed Hyperparameters of seven machine learning models are listed in **Supplementary Table 3**.

Model performance was assessed using metrics including area under the curve (AUC), accuracy, precision, recall, F1 score, and Brier score. Calibration curves and decision curve analysis (DCA) were employed to evaluate model calibration and net benefit. Subgroup analyses were conducted to confirm model stability and generalizability.

### 2.7 Model interpretability

The explainability of the model is of great significance in tumor research (24). To assess the association and agreement between intratumoral and peritumoral predictions, correlation analyses were performed. The SHAP framework elucidated model interpretability via global feature rankings and force plots, enabling biological and clinical insights. Restrictive cubic spline analysis (RCS) was used to explore the nonlinear relationship between features and research outcomes.

### 2.8 Statistical analysis

All analyses were performed using R version 4.1.2 (http://www.r-project.org/, Austria) and MedCalc (version 15.2). The R packages employed for classifier construction—including “mlr3”, “glm”, “rms”, “glmnet”, “caret”, “kNN”, “e1071”, and “randomForest”—provided a comprehensive and efficient interface for implementing various machine learning algorithms. Continuous variables are presented as mean ± standard deviation (SD) if normally distributed, with comparisons made using the independent-sample t-test; otherwise, the nonparametric Mann–Whitney *U* test was applied. Categorical variables were compared using the chi-square test. A two-tailed p-value < 0.05 was considered statistically significant.

## 3 Results

### 3.1 Patients characteristics

Ultimately, the patients’ demographic data and clinicoradiological characteristics are summarized in **Table 1**. This study recruited 261 ESCC patients, which 183 ESCC patients were analyzed as the training cohort (52 poorly-differentiated ESCC, 28.42%) and 78 ESCC patients were analyzed as the validation cohort (24 poorly-differentiated ESCC, 30.77%). In the training cohort, the differences of AST, ALP, and GGT were statistically significant between poorly- and well- and moderately-differentiated ESCC (P<0.05). Meanwhile, the difference of only sex was statistically significant in the validation cohort (P<0.05). In addition to that, the patients’ baseline characteristics were no statistically significant differences in the training cohort and validation cohort (*P*>0.05).

**Table 1:** The baseline characteristics of the ESCC patients.

Variables	Training cohort (N = 183)			Validation cohort (N = 78)		
	Poorly-differentiated ESCC (n=52)	Well- and moderately- differentiated ESCC (n=131)	P values	Poorly-differentiated ESCC (n=24)	Well- and moderately- differentiated ESCC (n=54)	P values
Sex:			0.782			0.026
Female	4 (7.7%)	13 (9.9%)		5 (20.8%)	2 (3.7%)	
Male	48 (92.3%)	118 (90.1%)		19 (79.2%)	52 (96.3%)	
Age(years)	68.6 ± 9.11	68.8 ± 8.85	0.894	68.7 ± 7.33	69.9 ± 6.27	0.497
WBC (x10^9^/L) ≤6.46:			0.128			1.000
NO	23 (44.2%)	76 (58.0%)		13 (54.2%)	29 (53.7%)	
YES	29 (55.8%)	55 (42.0%)		11 (45.8%)	25 (46.3%)	
NEU (x10^9^/L) ≥3.86:			0.062			1.000
NO	24 (46.2%)	82 (62.6%)		14 (58.3%)	32 (59.3%)	
YES	28 (53.8%)	49 (37.4%)		10 (41.7%)	22 (40.7%)	
LYM (x10^9^/L) ≤1.94:			0.085			0.261
NO	3 (5.8%)	22 (16.8%)		1 (4.2%)	8 (14.8%)	
YES	49 (94.2%)	109 (83.2%)		23 (95.8%)	46 (85.2%)	
MO (x10^9^/L) ≤0.61:			0.485			1.000
NO	14 (26.9%)	44 (33.6%)		7 (29.2%)	15 (27.8%)	
YES	38 (73.1%)	87 (66.4%)		17 (70.8%)	39 (72.2%)	
PLT (x10^9^/L) ≤296.00:			0.249			0.595
NO	9 (17.3%)	35 (26.7%)		5 (20.8%)	16 (29.6%)	
YES	43 (82.7%)	96 (73.3%)		19 (79.2%)	38 (70.4%)	
ALB(g/L) ≤43.10:			0.067			1.000
NO	6 (11.5%)	33 (25.2%)		4 (16.7%)	10 (18.5%)	
YES	46 (88.5%)	98 (74.8%)		20 (83.3%)	44 (81.5%)	
GLOB(g/L) ≥31.20:			0.079			0.052
NO	27 (51.9%)	88 (67.2%)		23 (95.8%)	41 (75.9%)	
YES	25 (48.1%)	43 (32.8%)		1 (4.17%)	13 (24.1%)	
ALT(U/L) ≤15.00:			0.310			1.000
NO	20 (38.5%)	63 (48.1%)		11 (45.8%)	26 (48.1%)	
YES	32 (61.5%)	68 (51.9%)		13 (54.2%)	28 (51.9%)	
AST(U/L) ≥20.60:			0.029			0.985
NO	17 (32.7%)	68 (51.9%)		11 (45.8%)	23 (42.6%)	
YES	35 (67.3%)	63 (48.1%)		13 (54.2%)	31 (57.4%)	
ALP(U/L) ≤52.00:			0.020			0.487
NO	41 (78.8%)	121 (92.4%)		20 (83.3%)	48 (88.9%)	
YES	11 (21.2%)	10 (7.6%)		4 (16.7%)	6 (11.1%)	
GGT(U/L) ≤14.00:			0.021			0.178
NO	34 (65.4%)	108 (82.4%)		16 (66.7%)	45 (83.3%)	
YES	18 (34.6%)	23 (17.6%)		8 (33.3%)	9 (16.7%)	
CA125 ≤ 19.3:			0.112			1.000
NO	12 (23.1%)	48 (36.6%)		8 (33.3%)	18 (33.3%)	
YES	40 (76.9%)	83 (63.4%)		16 (66.7%)	36 (66.7%)	
CA153(kU/L) ≥6.50:			0.068			0.843
NO	9 (17.3%)	42 (32.1%)		5 (20.8%)	14 (25.9%)	
YES	43 (82.7%)	89 (67.9%)		19 (79.2%)	40 (74.1%)	
CA199(kU/L) ≥13.10:			0.079			0.193
NO	27 (51.9%)	88 (67.2%)		13 (54.2%)	39 (72.2%)	
YES	25 (48.1%)	43 (32.8%)		11 (45.8%)	15 (27.8%)	
Region of ESCC:			0.582			0.558
Below	13 (25.0%)	30 (22.9%)		7 (29.2%)	11 (20.4%)	
Middle	30 (57.7%)	85 (64.9%)		14 (58.3%)	38 (70.4%)	
Upper	9 (17.3%)	16 (12.2%)		3 (12.5%)	5 (9.2%)	
Maximum thickness diameter (cm)	1.63 ± 0.62	1.58 ± 0.66	0.648	1.79 ± 0.63	1.65 ± 0.72	0.396
Length of ESCC (cm)	5.00 ± 2.05	5.23 ± 2.06	0.496	5.27 ± 2.59	5.01 ± 1.89	0.662

WBC, white blood cell; NEU, neutrophil; LYM, lymphocyte count; MO, monocyte count; PLT, platelet count; ALB, albumin; GLOB, globularproteins; ALT, alanine aminotransferase; AST, aspartate aminotransferase; ALP, alkaline phosphatase; GGT, Gamma-glutamyl transferase; ESCC, esophageal squamous cell carcinoma.

### 3.2 Laboratory selection and radiomics feature selection

In the training cohort, the LASSO regression and tenfold cross-validation were used for screening clinical laboratory parameters, and were shown in **Supplementary Figure 2**. Importantly, 10 features were screened from 19 clinical laboratory parameters.

Initially, the radiomic features of the intratumor, peritumoral 0.3 cm and intra-peritumoral 0.3 cm with poor reproducibility and ICC<0.8 were eliminated. After a series of feature dimensionality reduction, the mRMR was gradually used to screen for the features with the highest redundancy. Subsequently, the XGBoost algorithm was used to retain the top 15 features with the highest contribution, and the LASSO and tenfold cross-validation were used to further screen features from 15 features. The detailed display of the feature filtering process was summarized in **Supplementary Figures 3**, **4**. Ultimately, the radiomics scores of IRs, PRs and IPRs were calculated based on 8, 9, and 11 radiomics features (**Supplementary Table 4**). Heat map analysis suggested that there was no significant correlation between the final radiomics features (**Supplementary Figure 5**). Notably, ENs was constructed by logistic regression based on the IRs, PRs and IPRs.

### 3.3 Independent risk predictors selection

In order to identify independent risk predictors, the univariate and multivariate logistic regression analyses were progressively employed for the ten clinical laboratory parameters and ENs (**Table 2**). Eventually, the results suggested that ENs (Odds Ratio [OR]=2.752; P<0.001), NEU (OR = 2.488; P = 0.039) and ALP (OR = 3.519; P = 0.044) were independent risk predictors for predicting poorly-differentiated ESCC.

**Table 2:** Univariable and multivariable Logistic regression analysis of variables in the training cohort.

Variables	Univariable Analysis		Multivariable Analysis	
	OR 95%CI	P values	OR 95%CI	P values
NEU (≥ 3.86, <3.86)	1.952 (1.019-3.740)	0.044	**2.488 (1.045-5.924)**	**0.039**
Lymphocyte (≤1.94, >1.94)	3.297 (0.942-11.535)	0.062		
Albumin (≤43.1, >43.1)	2.582 (1.011-6.594)	0.047	0.852 (0.272-2.675)	0.784
Globulin (≥ 31.2, <31.2)	1.895 (0.984-3.648)	0.056		
AST (≥ 20.6, <20.6)	2.222 (1.134-4.357)	0.020	2.047 (0.829-5.054)	0.120
ALP (≤ 52.0, >52.0)	3.246 (1.285-8.201)	0.013	**3.519 (1.036-11.949)**	**0.044**
GGT (≤ 14.0, >14.0)	2.486 (1.201-5.145)	0.014	2.055 (0.782-5.401)	0.144
CA125 (≤ 19.3, >19.3)	1.928 (0.923-4.026)	0.081		
CA153 (≥ 6.5, <6.5)	2.255 (1.006-5.052)	0.048	2.330 (0.754-7.200)	0.142
CA199 (≥ 13.1, <13.1)	1.895 (0.984-3.648)	0.056		
ENs	2.719 (1.933-3.824)	<0.001	**2.752 (1.883-4.021)**	**<0.001**

OR, odds ratio; CI, confidence interval; NEU, neutrophils count; AST, aspartate aminotransferase; ALP, alkaline phosphatase; GGT, gamma-glutamyl transferase; CA125, carbohydrate antigen 125; CA153, carbohydrate antigen 153; CA199, carbohydrate antigen 199; ENs, the stacking score; ESCC, esophageal squamous cell carcinoma. Bold feature was an independent risk factor for predicting poorly differentiated ESCC before surgery.

### 3.4 Model building and comparison

Adding radiomics of peritumoral 0.3 cm to intratumor could improve the AUC in training cohort (from 0.765 to 0.824) and validation cohort (from 0.750 to 0.792). Moreover, the DCA suggested that combined intratumor and peritumoral 0.3 cm could obtain more net benefit, and was summarized in **Supplementary Figure 6**.

Ultimately, based on the ENs, NEU and ALP, seven machine learning models were established to evaluate prediction performance. Moreover, the ENML was built by using a simple logistic regression on the prediction probabilities of all machine learning models. The performance of eight models are summarized in **Table 3**. Importantly, ENML achieved the highest AUCs (0.865 *vs.* 0.799), the smallest Brier scores (0.134 *vs.* 0.166), an better F1 score (0.871 *vs.* 0.810) and an better precision (0.843 *vs.* 0.758) in training cohort and validation cohort.

**Table 3:** Comparison of the diagnostic performance of seven machine learning models and ENML in training cohort and validation cohort.

Models	AUC (95%CI)	Accuracy	Precision	Recall	Brier score	F1 score
Training Cohort						
RF	0.826 (0.763-0.878)**#**	0.771	0.783	**0.945**	0.152	0.856
LR	0.856 (0.796-0.903)**#**	0.787	0.816	0.903	0.139	0.857
KNN	0.805 (0.740-0.860)&	0.754	0.814	0.851	0.159	0.832
XGBoost	0.778 (0.710-0.836)&	0.715	0.791	0.818	0.205	0.804
SVM	0.800 (0.734-0.855)&	0.754	0.793	0.893	0.157	0.840
Bayes	0.821 (0.758-0.874)&	0.721	0.756	0.911	0.181	0.826
NNet	0.815 (0.751-0.868)&	0.760	0.833	0.835	0.157	0.834
ENML	**0.865 (0.807-0.911)**	**0.809**	**0.843**	0.901	**0.134**	**0.871**
Validation Cohort						
RF	0.728 (0.616-0.823)**#**	0.705	0.718	**0.944**	0.184	0.816
LR	0.792 (0.686-0.876)**#**	0.718	0.735	0.926	0.178	**0.819**
KNN	0.746 (0.634-0.838)**#**	0.679	0.723	0.870	0.207	0.790
XGBoost	0.778 (0.669-0.864)**#**	0.717	**0.767**	0.852	0.208	0.807
SVM	0.738 (0.626-0.831)**#**	0.692	0.721	0.907	0.199	0.803
Bayes	0.744 (0.632-0.836)**#**	0.679	0.716	0.889	0.221	0.793
NNet	0.716 (0.603-0.813)**#**	0.667	0.700	0.907	0.235	0.790
ENML	**0.799 (0.693-0.881)**	**0.719**	0.758	0.870	**0.166**	0.810

RF, random forest; LR, logistic regression; KNN, k-nearest neighbor; XGBoost, extreme gradient boosting classifiers; SVM, support vector machine; NNet, neural network; ENML, the ensemble model using a simple logistic regression of all machine learning models; AUC, area under the curve; CI, confidence interval.

^&^Compared with the ENML, there was statistically significant difference (DeLong test, P<0.05).

^**#**^Compared with the ENML, there was no statistically significant difference (DeLong test, P>0.05).

Bold value was the optimal one.

### 3.5 Model evaluation and validation

**Figure 2:**
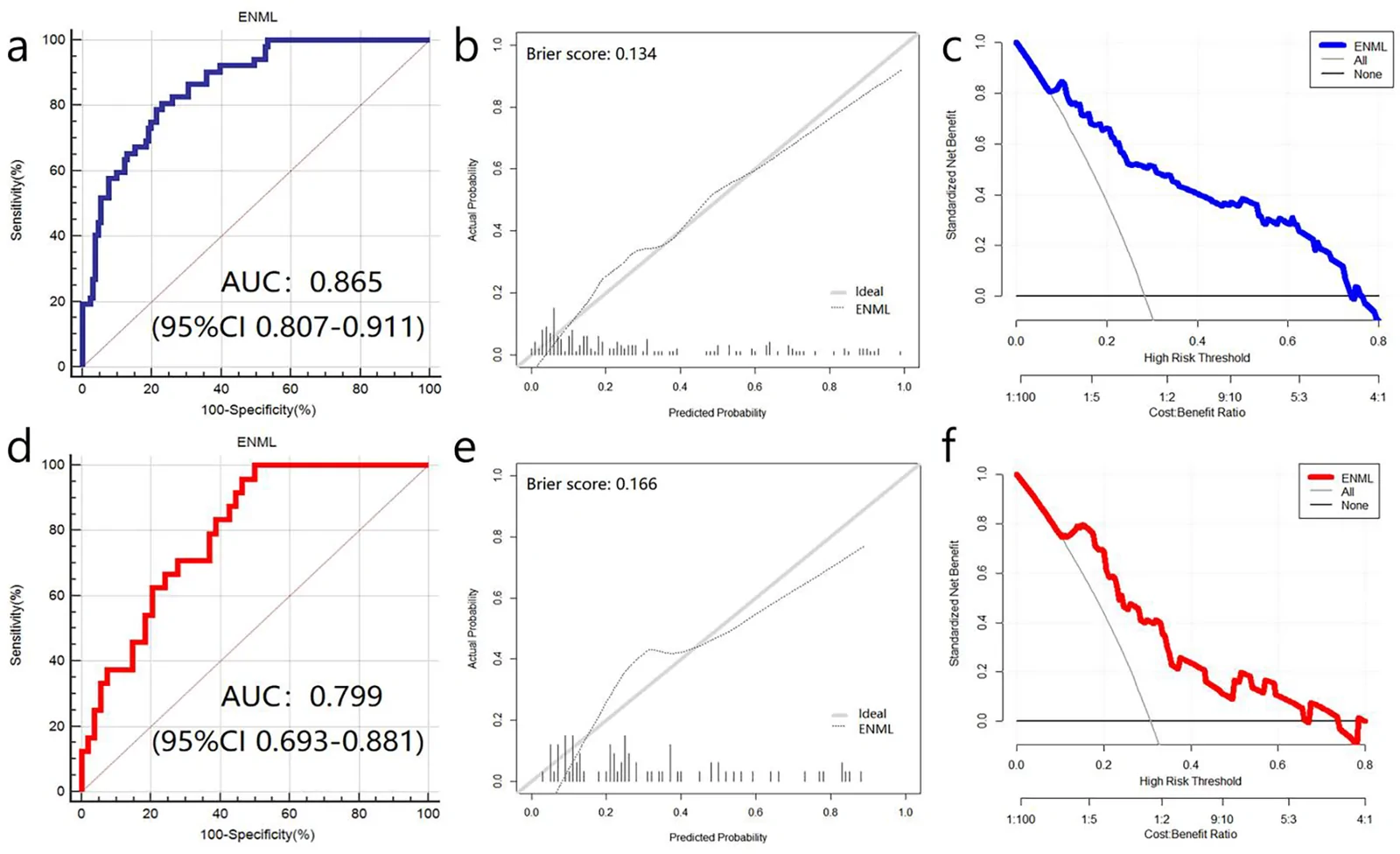
The ROC, calibration curve and DCA of the ENML for predicting the poorly differentiated ESCC in training cohort **(a–c)** and validation cohort **(d–f)**. ROC, receiver operating characteristic curve; DCA, decision curve analysis; ENML, the ensemble model using a simple logistic regression of all machine learning models.

The ENML owned an AUC of 0.865 (95% CI 0.807–0.911) in training cohort, an AUC of 0.799 (95% CI 0.693–0.881) in validation cohort, and was illustrated in **Figure 2**. The calibration curve suggested that ENML achieved good consistency with a Brier score of 0.134 in training cohort and a Brier score of 0.166 in validation cohort. DCA showed that ENML achieved more clinical net benefits within a wider threshold probability range from 10 to 75%.

Moreover, in the training cohort and validation cohort, the proportions of positive events were 28.42% and 30.77%, respectively. To account for the impact of class imbalance on the model, we conducted a balanced accuracy and precision-recall area under the curve (PR-AUC) analysis for the ENML. The results showed that the balanced accuracy of the ENML was 0.7907 and 0.7014 in the training cohort and validation cohort, with PR-AUC areas of 0.824 and 0.722, indicating that the model still maintains good discriminative capability despite class imbalance. The PR-AUC analysis was summarized in **Supplementary Figure 7**.

### 3.6 Bootstrap resampling and subgroup analysis

**Figure 3:**
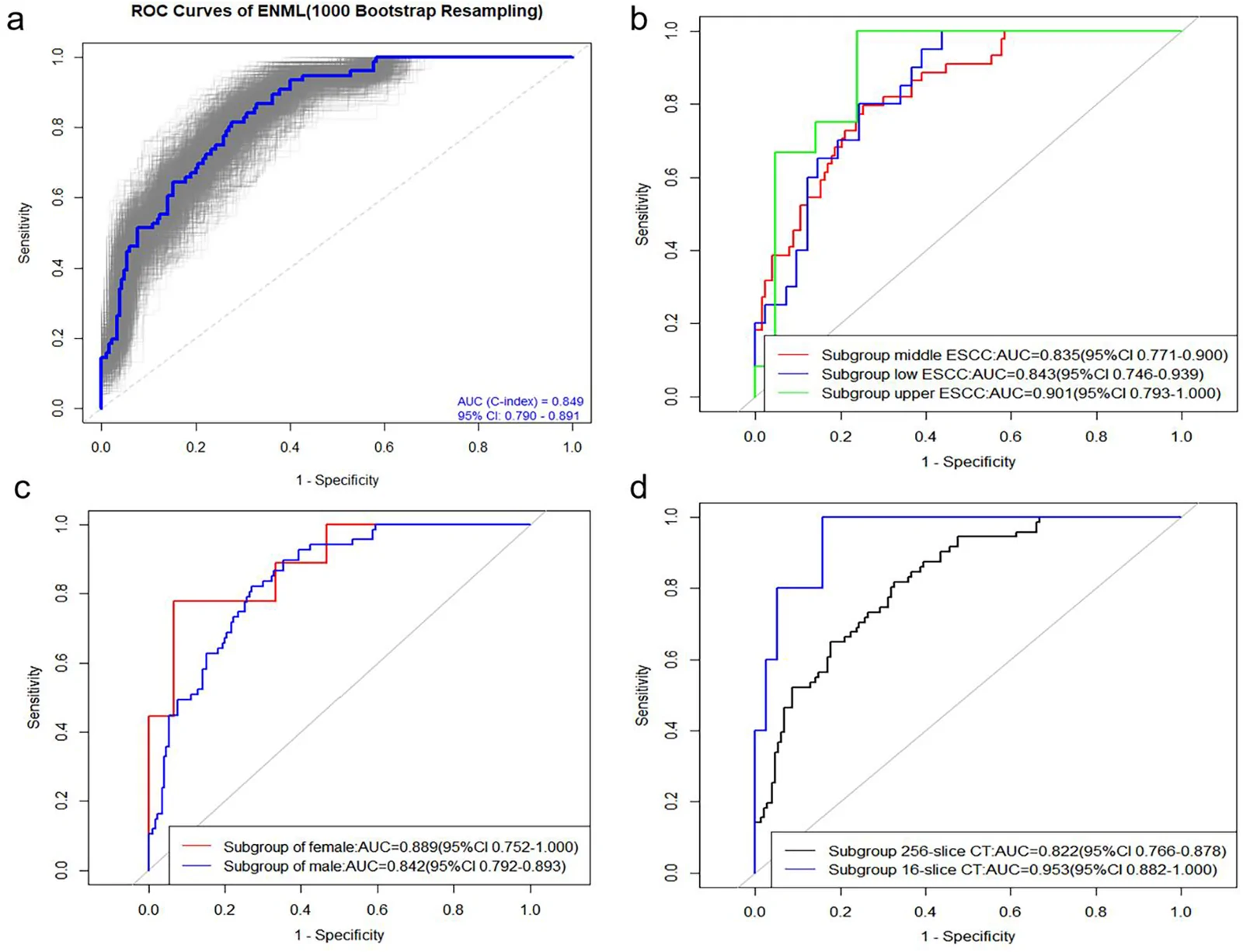
Bootstrap resamplings and subgroup analysis in full data cohort. After 1000 resamplings using Bootstrap **(a)**, ENML demonstrated stable diagnostic performance for predicting poorly differentiated ESCC, with an AUC of 0.849 (95%CI 0.790-0.891). Subgroup analysis of the ENML in different location of ESCC **(b)**, genders **(c)**, and CT equipment **(d)**. Abbreviations: ROC, receiver operating characteristic curve; ENML, the ensemble model using a simple logistic regression of all machine learning models; ESCC, esophageal squamous cell carcinoma.

To evaluate the accuracy and stability of ENML, we conducted Bootstrap 1000 resampling and subgroup analysis in the full data cohort. Importantly, after 1000 resamplings using Bootstrap, ENML demonstrated stable diagnostic performance for predicting poorly differentiated ESCC, with an AUC of 0.849 (95%CI 0.790-0.891). The ROC curve of the 1000 resampling of Bootstrap is summarized in **Figure 3a**.

Moreover, in order to investigate the stability of ENML in different CT scanners and different populations, subgroup analysis was employed for analysis. **Figures 3b–d** suggests that the ENML achieved a better and stable performance, with an AUC range of 0.822-0.953 in different genders [Male (n=237) *vs.* Female (n=24)], location of ESCC [Upper (n=33) *vs.* Middle (n=167) *vs.* Low (n=61)], and CT equipment [256-slice (n=218) *vs.* 16-slice (n=43)].

### 3.7 Model interpretability analysis

**Figure 4:**
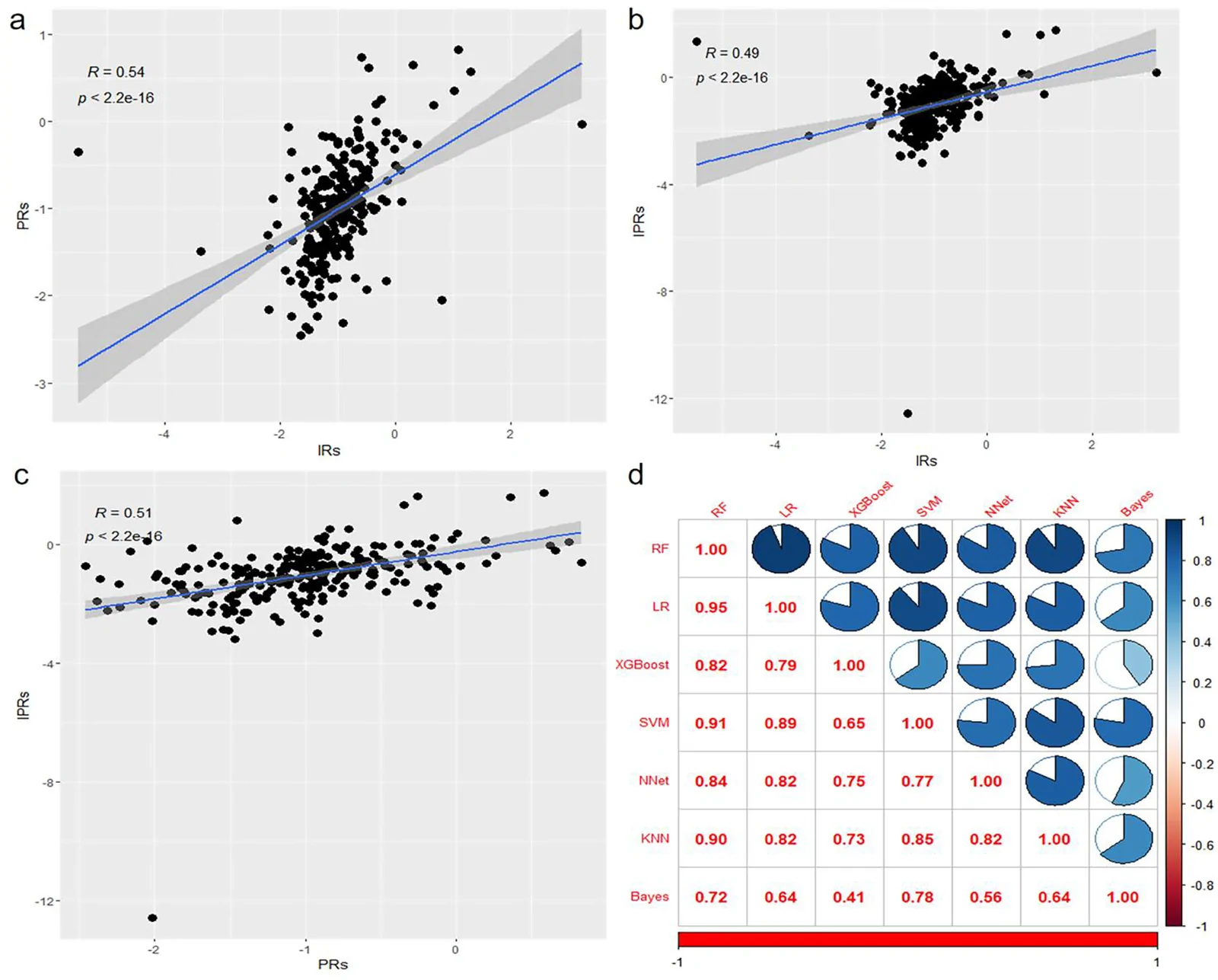
Correlation analysis. The scatter plot suggests a significant positive correlation between IRs and PRs **(a)**, between IRs and IPRs **(b)**, between PRs and IPRs **(c)**. Heatmap analysis of 7 machine learning models **(d)**. Abbreviations: IRs, the radiomics score of intratumor; PRs, the radiomics score of peritumoral 0.3 cm; IPRs, the radiomics score of intra-peritumoral 0.3 cm; RF, random forest; LR, logistic regression; KNN, k-nearest neighbor; XGBoost, extreme gradient boosting classifiers; SVM, support vector machine; NNet, neural network.

Correlation analysis was performed to investigate the relationship among intratumoral, peritumoral 0.3 cm, and intra-peritumoral 0.3 cm radiomics features. As shown in **Figures 4a–c**, the peritumoral and intratumoral radiomics exhibited strong positive correlations (Spearman’s r: 0.54, 0.49, 0.51; all P < 0.001), indicating synergistic effects and consistent predictive information for predicting the poorly-differentiated ESCC. Importantly, correlation heatmap analysis (**Figure 4d**) revealed positive associations among seven machine learning algorithms, suggesting that machine learning contributes collaboratively to the fusion model.

**Figure 5:**
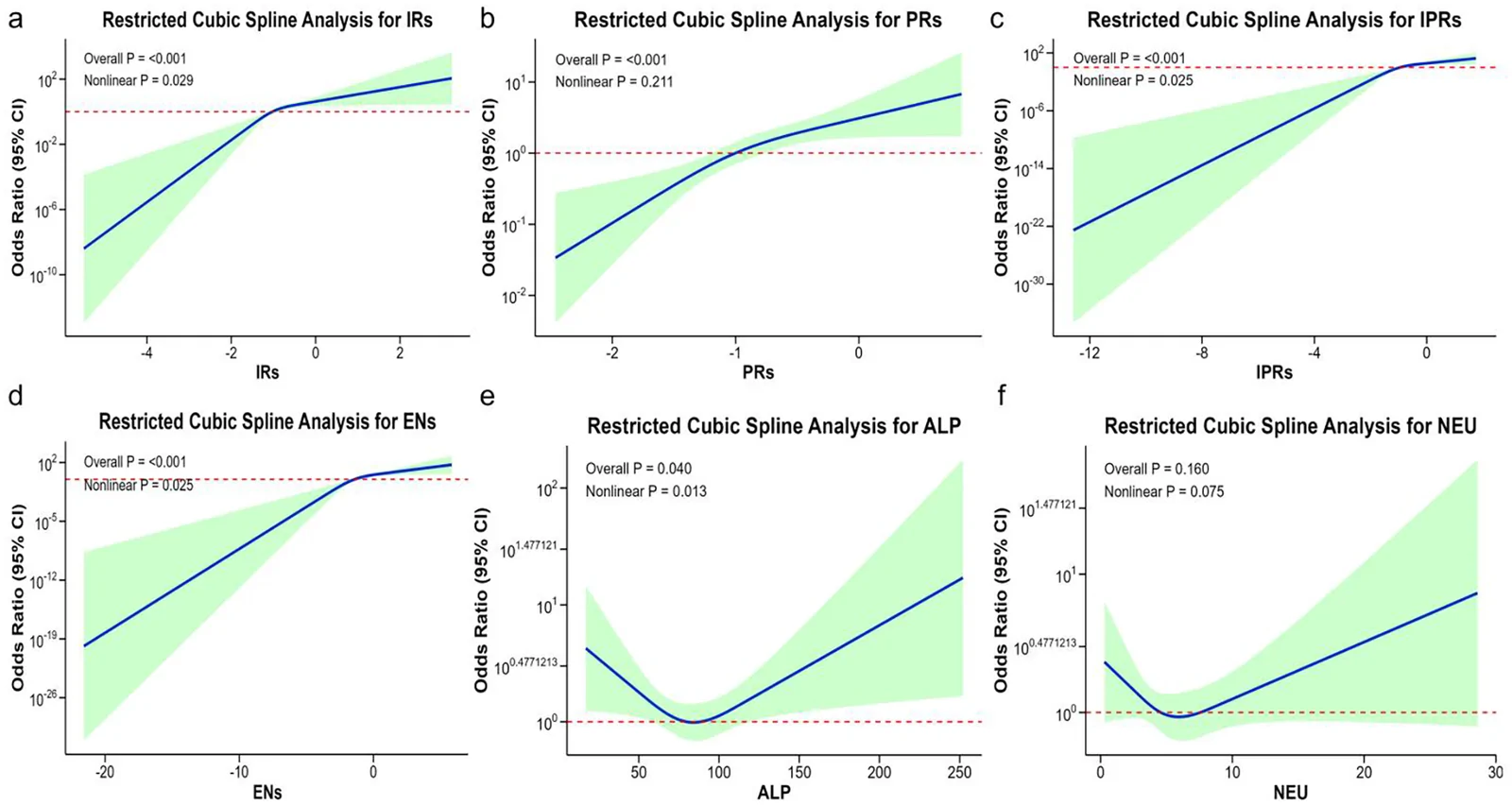
Restrictive cubic spline analysis for IRs **(a)**, PRs **(b)**, IPRs **(c)**, ENs **(d)**, ALP **(e)** and NEU **(f)**. The results indicate that IRs, IPRs, ENs, and ALP exhibit significant nonlinear relationships in preoperative prediction of poorly differentiated ESCC (Nonlinear P<0.05; Overall P<0.05), while ALP and NEU demonstrate significant inflection point effects. IRs, the radiomics score of intratumor; PRs, the radiomics score of peritumoral 0.3 cm; IPRs, the radiomics score of intra-peritumoral 0.3 cm; ENs, the stacking score of IRs, PRs, and IPRs; NEU, neutrophil; ALP, alkaline phosphatase.

RCS analysis showed a significant nonlinear correlation (Nonlinear P<0.05; Overall P<0.05) between IRs, IPRs, ENs, and ALP and the increased incidence of poorly differentiated ESCC; However, no nonlinear relationship was observed between PRs and NEU. As shown in **Figure 5**, when these indicators are at a lower level, they are associated with a decrease in the incidence of poorly differentiated ESCC; However, as the values of these indicators increase, the ratio gradually increases. Moreover, the ALP and NEU achieved significant inflection point effects in preoperative prediction of poorly differentiated ESCC. These findings may suggest that integrating peritumoral and intratumoral CT radiomics with serum indicators—potentially reflecting tumor–microenvironment interactions and the average value of systemic serum tumor burden—played a dominant role in enhancing the predictive accuracy of the fusion model.

**Figure 6:**
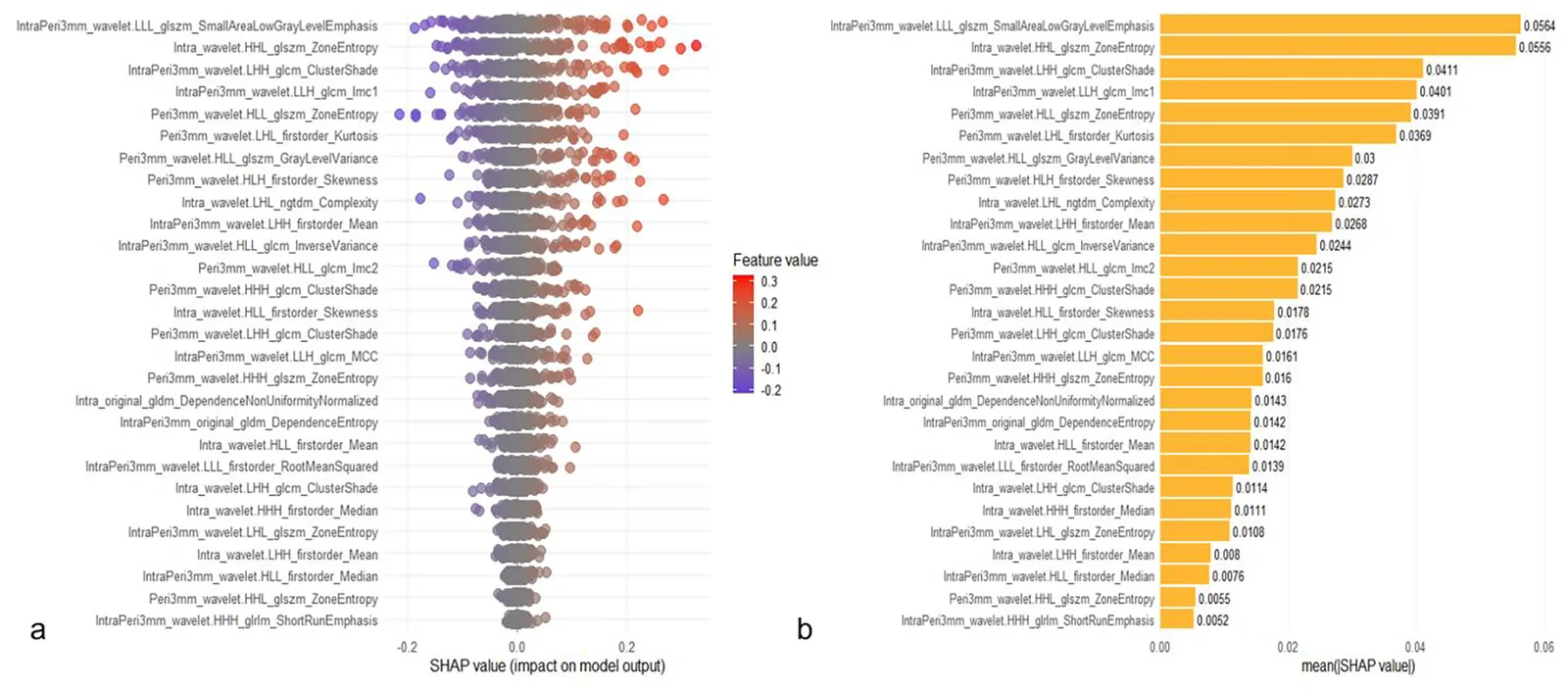
The swarm plot **(a)** and bar chart **(b)** of the SHAP analysis. SHAP analysis suggests that the IntraPeri3mm_wavelet.LLL_glszm_SmallAreaLowGrayLevelEmphasis contributed the most to the model, with an average Shapley value of 0.0564. SHAP, shapley additive exPlanations.

**Figure 7:**
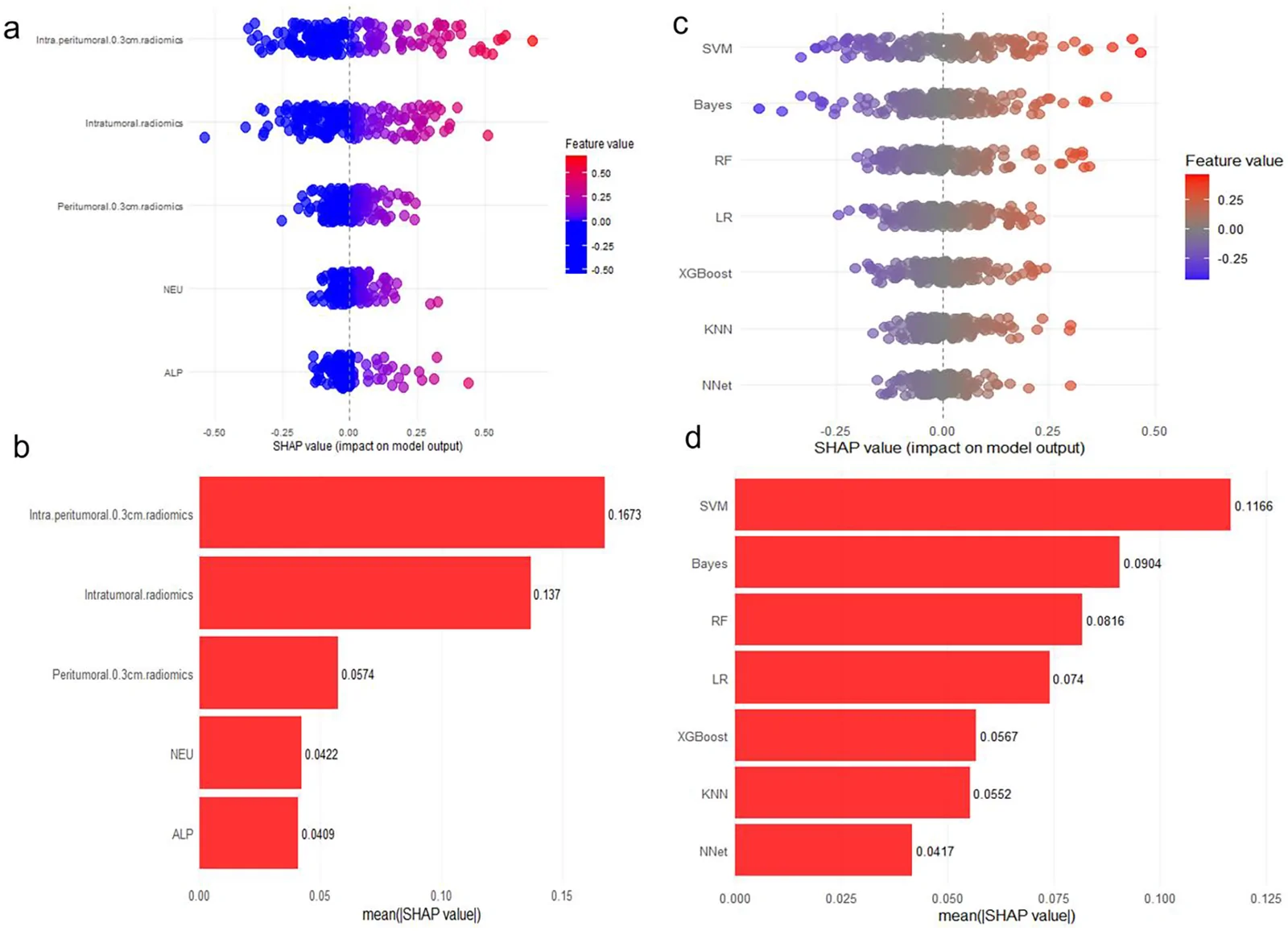
The swarm plot **(a, c)** and bar chart **(b, d)** of the SHAP analysis. SHAP analysis suggests that the intra-peritumoral 0.3 cm regions radiomics exhibited the greatest contribution, with a mean Shapley value of 0.1673 **(b)**. The machine learning of SVM achieved the greatest contribution, with an average Shapley value of 0.1166 **(d)**. SHAP, shapley additive exPlanations; RF, random forest; LR, logistic regression; KNN, k-nearest neighbor; XGBoost, extreme gradient boosting classifiers; SVM, support vector machine.

SHAP could enhance the interpretation and visualization of the importance of each feature in the optimal model. Among the features, the radiomics feature IntraPeri3mm_wavelet.LLL_glszm_SmallAreaLowGrayLevelEmphasis contributed the most, with a mean Shapley value of 0.0564 (**Figures 6a, b**), supporting the synergistic contribution and theoretical rationale for combining intratumoral and peritumoral 0.3 cm regions in the model. Similarly, the intra-peritumoral 0.3 cm regions radiomics exhibited the greatest contribution, with a mean Shapley value of 0.1673 (**Figures 7a, b**), revealing that integrating intratumoral and peritumoral radiomics exerted a synergistic enhancement on model performance. Notably, in the final ensemble model, the machine learning of SVM made the greatest contribution, with a Shapley value mean of 0.1166 (**Figures 7c, d**).

## 4 Discussion

We innovatively developed the ENML, which integrates seven machine learning, intratumoral and peritumoral CT radiomics, NEU and ALP, and demonstrated good performance for predicting the poorly differentiated ESCC with a good calibration. Correlation analyses suggested that the intratumoral and peritumoral radiomics exhibited strong positive correlations for predicting the poorly differentiated ESCC. RCS analysis showed a significant nonlinear correlation between IRs, IPRs, and ALP and the increased incidence of poorly differentiated ESCC; Moreover, the ALP and NEU achieved significant inflection point effects in predicting the poorly differentiated ESCC. The IntraPeri3mm_wavelet.LLL_glszm_SmallAreaLowGrayLevelEmphasis and SVM contributed the most for ENML from SHAP analyses. These findings may indicate that ENML has certain interpretability and good ability to distinguish pathological grading of ESCC.

Notably, our study has firstly found that neutrophils and ALP are independent risk factors for predicting poorly differentiated ESCC. Coffelt SB (25) suggested that tumor-associated neutrophils (TANs) promote tumor cell proliferation and invasion through the release of inflammatory mediators such as IL-6 and TNF-α, contributing to tumor hypo-differentiation. Furthermore, previous studies have shown that high MMP-9 expression is strongly associated with tumor differentiation (25, 26). Neutrophils also exert immunosuppressive effects by releasing arginase 1 (ARG1) and reactive oxygen species (ROS), which inhibit T-cell function and help tumor cells evade immune surveillance, particularly in poorly differentiated tumors (27). ALP, an enzyme widely expressed in human tissues, has been linked to tumor differentiation in various malignancies (28). Meanwhile, previous studies have shown that ALP expression is significantly higher in ESCC compared to normal esophageal tissues, with levels inversely correlated with tumor differentiation (29). The above research provides a potential theoretical basis for our findings. It is worth noting that neutrophils and ALP in serum reflect the average inflammatory state of the body over a period of time.

From a biological perspective, the complementary value of the intratumoral and peritumoral regions can be attributed to their distinct pathophysiological significance (13). Radiomic features within the tumor primarily reflect intratumoral heterogeneity in ESCC—such as necrosis, cell density, and vascular irregularity—which are closely linked to tumor pathological grade and aggressiveness (14). In contrast, radiomic features from the peritumoral region capture subtle alterations in the adjacent stroma, including inflammatory cell infiltration, fibroblast activation, micrometastasis, and early lymphovascular invasion (14, 15). Moreover, intratumoral radiomics focuses on imaging features within the tumor, such as texture, morphology, and intensity, which reflect tumor heterogeneity and biological behavior (30). Specifically, previous studies have shown that intratumoral radiomics features are closely associated with tumor differentiation (31, 32). For instance, poorly differentiated tumors exhibit higher heterogeneity, with elevated entropy values, gray-level co-occurrence matrix parameters, and histogram features compared to well-differentiated tumors (32). In our study, it was worth noting that 8, 9, and 11 radiomic features from intratumor, peritumoral 0.3 cm and intra-peritumoral 0.3 cm were independent predictive biomarkers for predicting poorly differentiated ESCC, and most of the features come from the wavelet features of the first order, glszm, glcm, glrlm and SmallAreaLowGrayLevelEmphasis. These features effectively reveal the biological characteristics of tumor cell proliferation, necrosis, and angiogenesis (33).

Interestingly, we discovered that adding radiomic of peritumoral 0.3 cm to intratumor could improve the AUC in training cohort and validation cohort. This also indicated that peritumoral radiomics reflected changes in the tumor microenvironment and invasiveness (33, 34); Thus, the poorly differentiated ESCC often exhibit pronounced peritumoral infiltration, inflammatory responses, and angiogenesis, with more aggressive. Our findings were similar to those of previous studies that radiomics features such as wavelet features, texture (e.g., gray-level inhomogeneity of the peritumour region), and morphological parameters (e.g., peritumoral infiltration extent) correlated significantly with tumor differentiation (33, 34). Importantly, the intratumour radiomics captures the heterogeneous features within the tumor, while peritumour radiomics analyses the alterations in the tumor microenvironment. Notably, correlation analysis also further revealed that the intratumoral and peritumoral CT radiomics captured strongly consistent information in predicting poorly differentiated ESCC (P < 0.001), which further demonstrated the predictive value of the peritumoral 0.3 cm zone band for the peritumoral microenvironment of poorly differentiated ESCC. Theoretically, the peritumoral 0.3 cm may represent the microenvironment at the invasive front of ESCC. The malignant tumor cells continuously remodel their surrounding matrix during the progression process, inducing infiltration of inflammatory cells, activation of fibroblasts, formation of micrometastatic foci, and early vascular invasion. Our results show a significant positive correlation between the intratumoral and peritumoral radiomics features, suggesting that they are not simply redundant but reflect different dimensions of the same biological process that are interrelated. The combination of these two can provide a more comprehensive assessment of the overall malignant potential of poorly differentiated ESCC. The ranking of feature importance based on SHAP values further confirms that the IntraPeri3mm_wavelet.LLL_glszm_SmallAreaLowGrayLevelEmphasis feature contributes the most, providing a theoretical basis for the combined application strategy. The possible reason for the analysis may be: The poorly differentiated ESCC cells are densely arranged with a high nuclear cytoplasmic ratio, and can appear as numerous small and relatively low-density regions on enhanced CT, which are precisely captured by this feature. The wavelet LLL sub-band highlights low-frequency information (i.e. relatively uniform and flat parts in the image), which may correspond to necrotic areas, cell rich dense areas, or mucinous degeneration areas inside the tumor. These pathological changes are more common in poorly differentiated ESCCs. This feature contributes the most in the 0.3 cm joint area within and around the tumor, indicating that poorly differentiated ESCC not only affect the tumor core, but also alter the surrounding microenvironment (such as edema, inflammation, and microinvasion). Importantly, the peritumoral 0.3 cm CT radiomics features capture the key pathological physiological changes in the tumor microenvironment and form a synergistic complementation with the intratumoral radiomics features, providing reliable imaging biomarkers for preoperative comprehensive assessment of poorly differentiated ESCC and facilitating individualized treatment decision-making.

Importantly, RCS analysis showed that there was a significant nonlinear correlation between IRs, IPRs, ENs and ALP and the increase of incidence rate of poorly differentiated ESCC. This finding may suggest that stacking radiomics of intratumor, and peritumoral 0.3 cm as well as serum biomarkers (which may reflect the interaction between the tumor and the microenvironment and the average value of systemic serum tumor burden) plays a dominant role in enhancing the predictive accuracy of the fusion model. Subgroup analysis suggested that the ENML achieved a better and stable performance, with an AUC range of 0.822-0.953 in different subgroup. However, in the subgroup (CT equipment: 16 slice), the AUC was as high as 0.953, and this result should be interpreted with caution. The reason may be that among the 43 samples, there were only 5 cases of poorly differentiated ESCC, indicating that the model is susceptible to the influence of a small number of samples in the 16 slice CT equipment subgroup, and its actual discriminative ability may be overestimated. Ultimately, the ENML integrates different machine learning algorithms, which not only have optimal predictive value, but also good discriminative ability and stability; Thus, this model may offer potential for guiding personalized surgical decisions.

Encouragingly, we envision that ENML may be embedded into clinical decision support systems through the “auxiliary screening and warning mode” and “individualized risk stratification and treatment recommendation mode”. The patient groups that may benefit the most are the following three categories: (1) ESCC patients with limited biopsy sampling or risk of sampling errors; (2) ESCC patients who cannot tolerate deep biopsy due to complications such as bleeding and stenosis; (3) Patients with early esophageal cancer (T1a/T1b) who are scheduled for endoscopic resection. ENML can help screen individuals who require careful evaluation before endoscopic resection, avoiding improper treatment due to underestimation of differentiation degree. However, it should be frankly stated that the above application ideas are still in the theoretical stage. Therefore, several limitations of our study should be acknowledged. First, this was a single-center retrospective study with a relatively small sample size, which may introduce selection bias. The clinical applicability of this model still needs to be further validated through prospective, multicenter, and large sample studies. Second, our analysis was limited to CT images acquired in the arterial phase. Although arterial-phase contrast-enhanced CT provides superior visualization of tumor microvascular heterogeneity and intratumoral microcirculatory perfusion (35, 36), the potential added value of incorporating non-contrast CT and venous-phase CT images warrants further investigation. Furthermore, we chose radiomics with a tumor margin of 0.3 cm. However, there is currently a lack of direct and systematic comparative evidence for different distances to rigorously demonstrate whether the 0.1 cm, 0.5 cm, or more tumor margin has supplementary value. This is the direction for future efforts. Third, converting continuous variables to binary variables may result in information loss and differences in truncation values. In the future, better strategies for handling continuous variables and multi center validation should be explored. Finally, this study did not explore the relationship between radiomics and the genomic landscape underlying ESCC pathological differentiation, nor did it include long-term follow-up data for the 261 enrolled patients. The robustness of the proposed model requires further validation in prospective, multicenter studies across diverse geographic regions and populations.

## 5 Conclusions

The ENML, which integrates seven different machine learning algorithms, intra-/peritumoral radiomics and serum biomarkers, demonstrates good discriminative capability in predicting the poorly differentiated ESCC. Correlation analyses suggested that the intratumoral and peritumoral radiomics exhibited strong positive correlations. RCS analysis showed a significant nonlinear correlation between IRs, IPR, and ALP and the increased incidence of poorly differentiated ESCC. The peritumoral 0.3 cm may represent the microenvironment at the invasive front of ESCC, and the IntraPeri3mm_wavelet.LLL_glszm_SmallAreaLowGrayLevelEmphasis made the greatest contribution for the final ensemble model. However, given that this study is a small-sample retrospective analysis without external validation, the above findings warrant further confirmation.

## Data Availability

The original contributions presented in the study are included in the article/**Supplementary Material**. Further inquiries can be directed to the corresponding author.
