# Supplementary material for: Interpretable machine learning integrating intra-/peritumoral CT radiomics and serum indicators for predicting poorly differentiated esophageal squamous cell carcinoma
Source: Front Oncol. 2026 Sep 4;16:1839893. doi: 10.3389/fonc.2026.1839893 (PMC13585512; doi:10.3389/fonc.2026.1839893)
Supplement: Supplementary file 1 [file Table1.doc]

***Supplementary Material***

**1.Supplementary Tables**

**Supplementary Table 1**

The detailed laboratory parameters and optimal cut-off values for predicting poorly differentiated ESCC using ROC curve analysis.

| **Detailed laboratory parameters** | **Optimal Cutoff Values** |
| --- | --- |
| White blood cell count (x10^9^/L; WBC) | 6.46 |
| Neutrophil count (x10^9^/L; NEU) | 3.86 |
| Lymphocyte count (x10^9^/L; LYM) | 1.94 |
| Monocyte count (x10^9^/L; MO) | 0.61 |
| Platelet count (x10^9^/L; PLT) | 296.00 |
| Albumin (g/L; ALB) | 43.10 |
| Globularproteins (g/L; GLOB) | 31.20 |
| Alanine aminotransferase (U/L; ALT) | 15.00 |
| Aspartate aminotransferase (U/L; AST) | 20.60 |
| Alkaline phosphatase (U/L; ALP) | 52.00 |
| Gamma-glutamyl transferase (U/L; GGT) | 14.00 |
| CA125 (kU/L) | 19.30 |
| CA153 (kU/L) | 6.50 |
| CA199 (kU/L) | 13.09 |

Abbreviations: ROC, receiver operating characteristic; ESCC, esophageal squamous cell carcinoma.

**Supplementary Table 2**

**The scanning parameters of chest-enhanced CT scanning**

| Scan parameters | Revolution 256-slice spiral CT (*General GE, USA*) | LightSpeed 16-slice spiral CT  (*General GE, USA*) |
| --- | --- | --- |
| Patients (%) | N=218 (83.53%) | N=43 (16.47%) |
| Tube voltage (kVp) | 120 | 120 |
| Tube current (mA) | automatic | automatic |
| Gathering matrix | 512×512 | 512×512 |
| Pitch | 1.0 | 1.375 |
| Layer thickness(mm) | 2.5 | 5 |
| Reconstruction thickness(mm) | 1.25 | 1.25 |

Abbreviations: CT, computed tomography.

**Supplementary Table 3**

**The detailed Hyperparameters of seven machine learning models**

| **Models** | **Random Seed** | **Hyperparameters** | **Methods** |
| --- | --- | --- | --- |
| RF | Seed(123) | ntree = 500,   mtry = sqrt(ncol(training_set) - 1),   importance = TRUE,   proximity = FALSE,   na.action = na.omit,   replace = TRUE,   sampsize = nrow(training_set),   nodesize = 1,   maxnodes = NULL | Grid Search, 10-Fold Cross-Validation |
| LR | Seed(123) | family = "binomial",  alpha = alpha,  type.measure = "deviance",   nfolds = 10,   standardize = TRUE,   parallel = FALSE,   thresh = 1e-07,   maxit = 100000,   penalty.factor = rep(1, ncol(x_train)),   intercept = TRUE,   grouped = TRUE | Grid Search, 10-Fold Cross-Validation |
| KNN | Seed(123) | k = 15,   distance = 2,   kernel = "rectangular",   scale = TRUE,   use.all = TRUE,   prob = TRUE,   algorithm = "kd_tree",   tuneLength = 10,   cv_folds = 10 | Grid Search, 10-Fold Cross-Validation |
| XGBoost | Seed(123) | booster = "gbtree",   objective = "binary:logistic",   eval_metric = "logloss",   max_depth = 6,   eta = 0.3,   gamma = 0,   min_child_weight = 1,   subsample = 0.8,   colsample_bytree = 0.8,   lambda = 1,   alpha = 0,   nthread = 4 | Grid Search, 10-Fold Cross-Validation |
| SVM | Seed(123) | kernel = "radial",   cost = 1,   gamma = 0.5,   probability = TRUE,   scale = TRUE,   cross = 10,   cache_size = 100,   tolerance = 0.001,   epsilon = 0.1,   shrinking = TRUE,   class.weights = NULL | Grid Search, 10-Fold Cross-Validation |
| Bayes | Seed(123) | laplace = 1,   usekernel = TRUE,   adjust = 1,   fL = 0,   usekernal = TRUE,   prior = NULL,   threshold = 0.001,   eps = 0,   subset = NULL,   na.action = na.pass | Grid Search, 10-Fold Cross-Validation |
| NNet | Seed(123) | size = 5,   maxit = 200,   decay = 0.0001,   rang = 0.7,   skip = FALSE,   Hess = FALSE,   trace = TRUE,   MaxNWts = 1000,   abstol = 1.0e-4,   reltol = 1.0e-8,   learning_rate = 0.01,   activation = "logistic",   algorithm = "rprop+",   hidden_layers = 1,   batch_size = 10 | Grid Search, 10-Fold Cross-Validation |

Abbreviations: RF, random forest; LR, logistic regression; KNN, k-nearest neighbor; XGBoost, extreme gradient boosting classifiers; SVM, support vector machine; NNet, neural network

**Supplementary Table 4**

The detailed calculation and construction process of IRs, PRs, IPR and ENs.

| **Label** | **Feature Name** | **Coefficient values** |
| --- | --- | --- |
| IRs | Intercept | 2.4624299 |
| **IR1**  **IR2**  **IR3**  **IR4**  **IR5**  **IR6**  **IR7**  **IR8** | original_gldm_DependenceNonUniformityNormalized  wavelet.LHL_ngtdm_Complexity  wavelet.LHH_firstorder_Mean  wavelet.LHH_glcm_ClusterShade  wavelet.HLL_firstorder_Mean  wavelet.HLL_firstorder_Skewness  wavelet.HHL_glszm_ZoneEntropy  wavelet.HHH_firstorder_Median | 1.2673863  3.3552741  -0.1848500  -2.9425651  -1.0493118  -3.6616554  -1.2299095  0.7792394 |
| **IRs**=2.4624299+1.2673863***IR1**+3.3552741***IR2**-0.18485***IR3**-2.9425651***IR4**-1.0493118***IR5**-3.6616554***IR6**-1.2299095***IR7**+0.7792394***IR8** | | |
| **Label** | **Feature Name** | **Coefficient values** |
| PRs | Intercept | -2.2461896 |
| **PR1**  **PR2**  **PR3**  **PR4**  **PR5**  **PR6**  **PR7**  **PR8**  **PR9** | wavelet.LHL_firstorder_Kurtosis  wavelet.LHH_glcm_ClusterShade  wavelet.HLL_glcm_Imc2  wavelet.HLL_glszm_GrayLevelVariance  wavelet.HLL_glszm_ZoneEntropy  wavelet.HLH_firstorder_Skewness  wavelet.HHL_glszm_ZoneEntropy  wavelet.HHH_glcm_ClusterShade  wavelet.HHH_glszm_ZoneEntropy | 1.6858992  -3.1792054  -1.6110694  2.4923802  0.1429442  1.7055207  -0.5953172  1.6593131  0.3525928 |
| **PRs**=-2.2461896+1.6858992***PR1**-3.1792054***PR2**-1.6110694***PR3**+2.4923802***PR4**+0.1429442***PR5**+1.7055207***PR6**-0.5953172***PR7**+1.6593131***PR8**+0.3525928***PR9** | | |
| **Label** | **Feature Name** | **Coefficient values** |
| IPRs | Intercept | 7.6740850 |
| **IPR1**  **IPR2**  **IPR3**  **IPR4**  **IPR5**  **IPR6**  **IPR7**  **IPR8**  **IPR9**  **IPR10**  **IPR11** | original_gldm_DependenceEntropy  wavelet.LLH_glcm_Imc1  wavelet.LLH_glcm_MCC  wavelet.LHL_glszm_ZoneEntropy  wavelet.LHH_firstorder_Mean  wavelet.LHH_glcm_ClusterShade  wavelet.HLL_firstorder_Median  wavelet.HLL_glcm_InverseVariance  wavelet.HHH_glrlm_ShortRunEmphasis  wavelet.LLL_firstorder_RootMeanSquared  wavelet.LLL_glszm_SmallAreaLowGrayLevelEmphasis | -2.0838520  -0.5243947  0.3042595  -1.1010042  -0.4633900  -13.3580014  -1.5960570  -2.7232545  0.4612661  -0.4523308  -2.0069761 |
| **IPRs**=7.674085-2.083852***IPR1**-0.5243947***IPR2**+0.3042595***IPR3**-1.1010042***IPR4**-0.46339***IPR5**-13.3580014***IPR6**-1.596057***IPR7**-2.7232545***IPR8**+0.4612661***IPR9**-0.4523308***IPR10**-2.0069761***IPR11** | | |
| **Modle** | **Feature Name** | **Coefficient values** |
| ENs | Intercept | 1.464 |
| **EN1**  **EN2**  **EN3** | IRs  PRs  IPRs | 1.004  0.136  1.690 |
| **ENs** = 1.464+1.004***IRs**+0.136***PRs**+1.690***IPRs** | | |

Abbreviations: IRs, the radiomics score of intratumor; PRs, the radiomics score of peritumoral 0.3cm; IPRs, the radiomics score of intratumoral+peritumoral 0.3cm; ENs, the stacking score of IRs, PRs, and IPRs.

**2. Supplementary Figures**

**Supplementary Figure 1**

The flowchart of patient screening in the study.

**Supplementary Figure 2**

Clinical laboratory parameters selection path plots of LASSO regression (**a**) and tenfold cross-validation (**b**) in the training cohort. The black line represents the log(Lambda) value (Lambda=0.0449897) corresponding to the lowest number of variables within one standard error criteria of the binomial deviation, where ten clinical parameters were included.

**Supplementary Figure 3**

**A:**

Radiomics feature dimension reduction process of peritumoral 0.3cm based on XGBoost algorithm (**a**), LASSO (**b**) and ten-fold cross-validation (**c**).

**B:**

Radiomics feature dimension reduction process of intratumor based on XGBoost algorithm (**a**), LASSO (**b**) and ten-fold cross-validation (**c**).

**C:**

Radiomics feature dimension reduction process of intratumor+peritumoral 0.3cm based on XGBoost algorithm (**a**), LASSO (**b**) and ten-fold cross-validation (**c**).

**Supplementary Figure 4**

Flowchart of radiomics feature screening.

Abbreviations: ICC, interclass correlation coefcient; mRMR, maximum correlation and minimum redundancy; XGBoost, extreme gradient boosting classifiers; LASSO, least absolute shrinkage and selection operator.

**Supplementary Figure 5**

Heat map analysis of the final radiomics features. Intratumor (**a**), peritumoral 0.3cm (**b**), and intratumoral + peritumoral 0.3cm (**c**).

**Supplementary Figure 6**

Comparison the ROC and DCA curves of adding radiomic features of peritumoral 0.3cm to intratumor in training cohort (**a, c**) and validation cohort (**b, d**).

Abbreviations: IRs, the radiomics score of intratumor; PRs, the radiomics score of peritumoral 0.3cm; IPRs, the radiomics score of intratumoral+peritumoral 0.3cm; ROC, receiver operating characteristic; DCA, decision curve analysis.

**Supplementary Figure 7**

Analysis of Precision Recall Curve (PR-AUC Curve) for Category Imbalance. **a**, training cohort; **b**, validation cohort.
